# Molecular network-based analysis of the mechanism of liver injury induced by volatile oils from *Artemisiae argyi folium*

**DOI:** 10.1186/s12906-017-1997-4

**Published:** 2017-11-16

**Authors:** Hongjie Liu, Sha Zhan, Yan Zhang, Yan Ma, Liang Chen, Lingxiu Chen, Hanqiu Dong, Min Ma, Zhe Zhang

**Affiliations:** 1 Department of Traditional Chinese Medicine, Jinan University, Guangzhou, 510632 China; 2 Shandong University of Traditional Chinese Medicine, Jinan, 250355 China

**Keywords:** Volatile oils from *Artemisiae argyi folium*, Chinese herbal drugs, Drug-induced liver injury, Bioinformatics

## Abstract

**Background:**

Volatile oils from *Artemisiae argyi folium* (VOAAF) is reported with hepatotoxicity, but the underlying mechanism is still unclear.

**Methods:**

In the present study this molecular mechanism was explored with the Ingenuity Pathway Analysis (IPA). The chemical components of the VOAAF were searched in the database, and their target proteins were all identified in the PubChem, while drug-induced liver injury (DILI) genes were searched in the PubMed gene databases. The molecular network of protein targets for VOAAF and DILI genes was built with the IPA. The canonical pathways between the 2 networks were compared to decipher the molecular mechanisms of the liver injury induced by VOAAF.

**Results:**

There were 159 target proteins for VOAAF and 338 genes related to DILI identified, which were further analyzed in the IPA. The canonical pathway comparison showed that VOAAF and DILI both worked on aryl hydrocarbon receptor (AHR), lipopolysaccharide (LPS)/interleukin 1 (IL-1) mediated inhibition of retinoid X receptor (RXR) function, pregnane X receptor (PXR)/RXR activation, xenobiotic metabolism, peroxisome proliferator-activated receptor (PPAR), hepatic cholestasis, farnesoid X receptor (FXR)/RXR activation, and glucocorticoid receptor.

**Conclusion:**

VOAAF-induced liver injury may be involved in many pathways in which the AHR signaling and LPS/IL-1 mediated inhibition of RXR function pathways could be the most vital.

**Electronic supplementary material:**

The online version of this article (doi: 10.1186/s12906-017-1997-4) contains supplementary material, which is available to authorized users.

## Background

The concept of bioinformatics was for the first time introduced at the forum of Information Theory in Biology in 1956 in Gatlinburg, Tennessee, USA [1, 2]. Bioinformatics, a discipline, based on the study of the biological data, applies computer science, statistics, mathematics, and engineering to analyze and interpret biological data [3, 4]. Bioinformatics analysis technology, which can integrate multiple data and help us to better understand biological processes in organic life, has become an essential way for life sciences studies. With the progress and development of modern biological technology, bioinformatics has been applied to the research work in TCM combined with other biological disciplines. Chinese herbal drugs have many compositions and various pharmacological effects. In order to understand the underlying mechanism, currently bioinformatics has been applied to study the functions and biological properties of Chinese herbal drugs [5].

For example, anti-tumor mechanism of *Artemisia annua* was studied by using on bioinformatics, and the results showed that the anti-tumor effect of *Artemisia annua* may be associated with regulating lipid metabolism of tumor cells, releasing energy, reducing the rate of tumor cell division and accelerating tumor cell apoptosis [6]. Also bioinformatics was applied to study the anti-Alzheimer’s herbal medicines, and it was found that some herbal medicines were promising for treating Alzheimer’s disease [7]. Besides, screening using modular analysis bioinformatics techniques found 6 core functional modules for tumor-associated macrophages (TAMs) that contain 46 total genes [8].

*Artemisiae argyi folium*, the dry leaf of *Artemisia Argyi* Lévl. et Vant, is one of the commonly-used Chinese herbal drugs. According to theory of traditional Chinese medicine (TCM), *Artemisiae argyi folium* enters liver, spleen and kidney meridians, with warm property, pungent and bitter flavors and of slightly toxicity [9]. Reportedly, oral administration of 20–30 g of the crude drugs would result in acute liver injury or even death [10]. The chemical components of *Artemisiae argyi folium* mainly include volatile oils, flavonoid, tannins, triterpenes and polysaccharides. Volatile oils from *Artemisiae argyi folium* (VOAAF) have strong pharmacological activities and toxicity. The pharmacological activities of VOAAF include anti-inflammatory, antimicrobial, antiasthma, analgesic, antivirus, antioxidant, antitumor and immunomodulatory effects [11–14]. Although the toxicity of plants may also be the presence of heavy metals and secondary metabolites [15, 16], the hepatotoxicity of *Artemisiae argyi folium* is mainly from its volatile oils [17, 18], while the underlying mechanism of VOAAF leading to acute liver injury has not been clarified so far.

In the present study, drug-induced liver injury (DILI) and VOAAF were investigated through bioinformatics by searching public databases such as GenBank, PubChem, and China National Knowledge Infrastructure (CNKI).

## Methods

### DILI-related gene data

DILI related genes were searched in NCBI Gene database (http://www.ncbi.nlm.nih.gov/gene), which integrates information from a wide variety of species. A record may include nomenclature, reference sequences, maps, pathways, variations, phenotypes, and links to genome-, phenotype-, and locus- specific resources worldwide. We searched the Gene database (until June 1, 2016) with “drug induced liver injury” or “DILI” as keywords, DILI genes were targeted for *Homo sapiens*. (Additional file 1: Table S1).

### VOAAF-related target proteins

The target proteins of the VOAAF were searched in PubChem (http://pubchem.ncbi.nlm.nih.gov/) (until June 1, 2016), which is a public database on biological activities of small molecules and small interfering RNAs (siRNAs), hosted by the National Institutes of Health of American, consisting of three databases: PubChem Compound, PubChem Bioassay, and PubChem Substance. All the targeted proteins related to the active compounds in Chinese herbal drugs can be obtained from PubChem. After searching in the Grand Dictionary of Chinese Medicine [6], Chinese Materia Medical [7], Pharmacopoeia of the People’s Republic of China [9] and CNKI, the active compounds of the VOAAF were determined, and then all the determined compounds were searched in PubChem Compound. Because of the bio-information could be cross-referenced to other NCBI databases [8], the target proteins of active compounds that were also searched in PubChem. The protein category was limited to *Homo sapiens* (Additional file 1: Table S2).

### Networks built and analysis

**Fig. 1:**
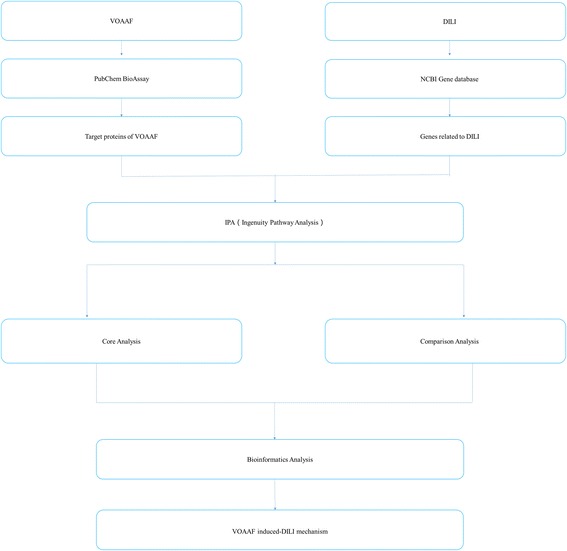
Frame diagram of the current research

Related DILI genes were input into the IPA software online (IPA, http://www.ingenuity.com), to build the molecular gene network (Fig. 1). Similarly, the VOAAF target proteins were input into IPA software online (IPA, http://www.ingenuity.com). The molecules input into IPA software were termed as “focus molecules”. IPA generated focus molecules protein-protein interaction (PPI) as a set of networks based on different bio-functions. Molecules were represented as nodes, and the biological relationship between two nodes is represented as an edge (line). All the edges were supported by at least 1 reference from the literature, textbooks, or from canonical information stored in the Ingenuity Knowledge Base. Nodes were displayed using various shapes that represent the gene product. The networks were evaluated by the scores, which were generated through calculation in the IPA software and represented the significance of the molecules in the network. To investigate the mechanism of DILI induced by the VOAAF, a canonical pathway analysis was performed using the IPA compare module. Through Fisher’s exact test, the significance of the association between focus molecules and the canonical pathways were determined in the IPA software.

## Results

### DILI-related gene networks and VOAAF-related target protein networks

**Fig. 2:**
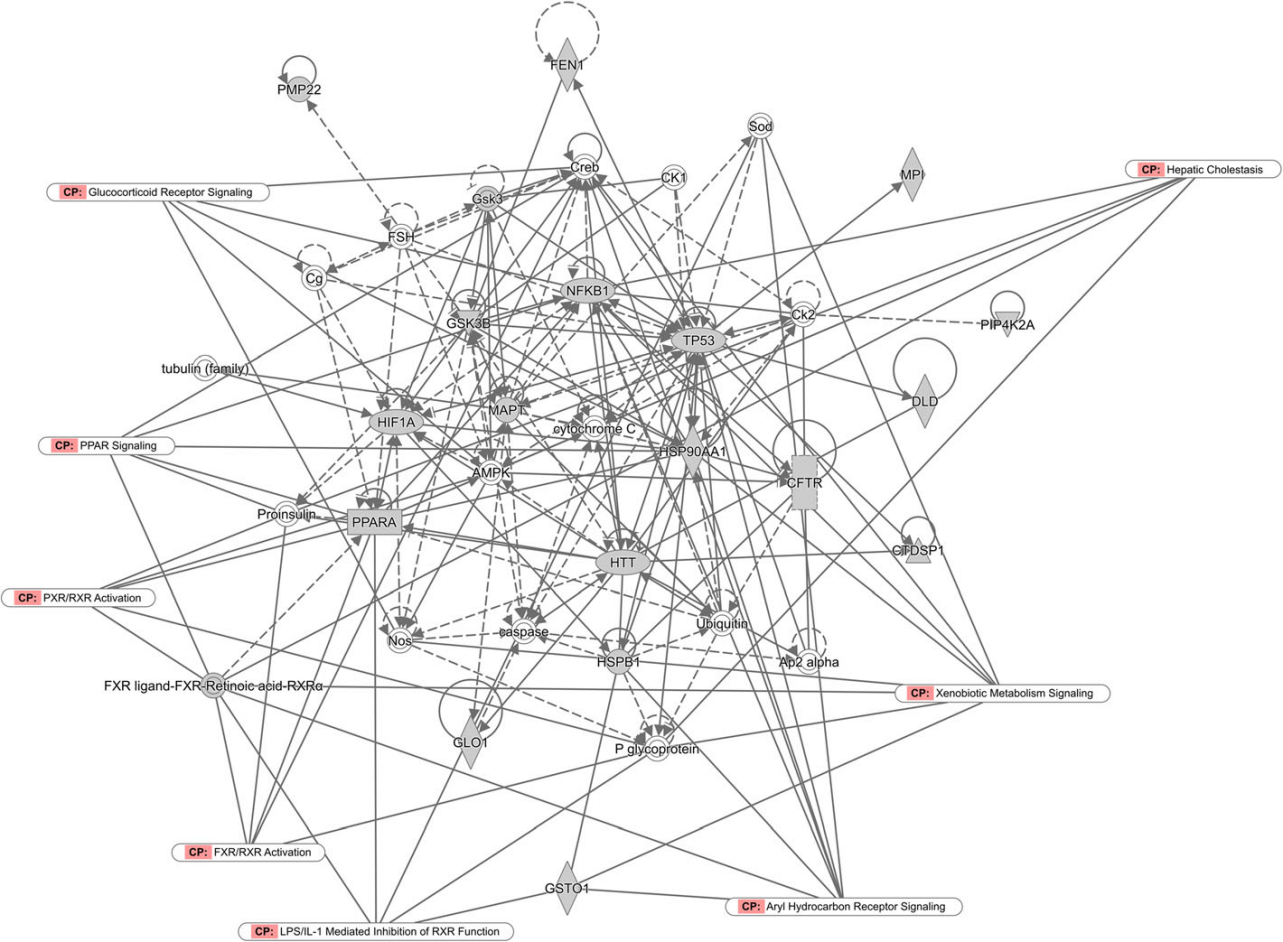
The molecular network of DILI

In this study, genes related to DILI were determined and the mechanism of the VOAAF-induced liver injury were explored by using a complex bioinformatics system correlated with GenBank database and target proteins. In the GenBank database 338 genes were identified (Additional file 1: Table S1) involved in DILI. By using the Ingenuity Pathway Analysis (IPA) kit (QIAGEN, Shanghai, China), the DILI molecular network was determined (Fig. 2) by importing the associated information for the 338 genes. As shown in Fig. 2, the most related pathways to DILI gene were overlapped. The main functions of the DILI molecular network were cellular function and maintenance, cell death and survival, cell-to-cell signaling and interaction.

**Fig. 3:**
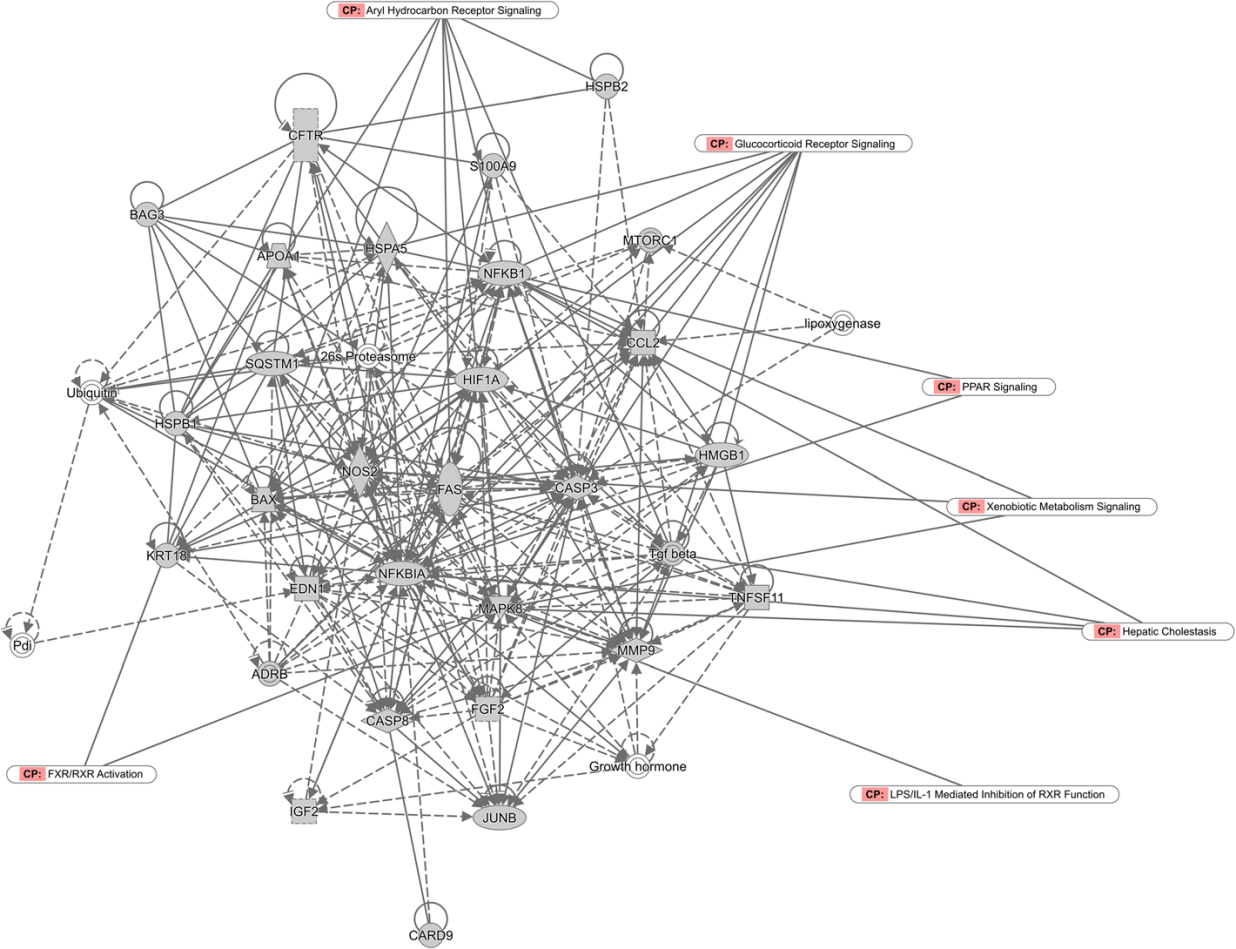
The molecular network of VOAAF

After searched in the PubChem database, information on the drug targets of the VOAAF were listed in Additional file 2: Table S2. The Gen Info Identifier (GI) numbers for each protein were imported into the IPA software, and the PPI networks of VOAAF were constructed (Fig. 3). As shown in Fig. 3, the most related pathways to VOAAF proteins were overlapped. The VOAAF target proteins were involved in metabolic disease, neurological disease, organismal injury and abnormalities.

From the comparison analysis it was found that both the DILI and VOAAF networks, as well as the functions of related target proteins were related on the cell death and survival, cell cycle, lipid metabolism, small molecule biochemistry, vitamin and mineral metabolism.

### Diseases and toxic functions

**Fig. 4:**
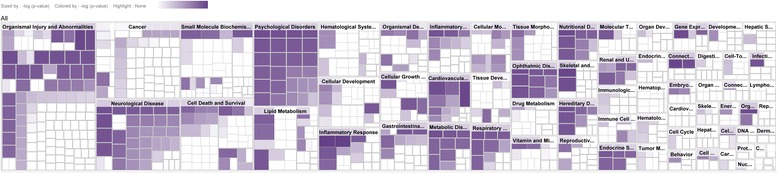
The heat map of diseases and toxic functions related to target proteins of VOAAF

The functional analysis identified the toxic functions and/or diseases that were most significant to the data set. Proteins from the data set that met the *P* value threshold of 0.05 (Fisher’s exact test) and were associated with toxic functions and/or diseases in the Ingenuity Pathway Analysis Knowledge Database were analyzed. From the IPA analysis (Fig. 4), it was found that the target proteins of VOAAF involved in the diseases and toxic functions are as follows: failure of heart, chronic heart failure, cell death of kidney cell lines, damage of liver, apoptosis of kidney cell lines, injury of liver, hepatocellular carcinoma, necrosis of liver, inflammation of liver, viral hepatitis, chronic viral hepatitis and proliferation of liver cells.

### The canonical pathway of DILI and VOAAF networks analyzed by IPA

**Fig. 5:**
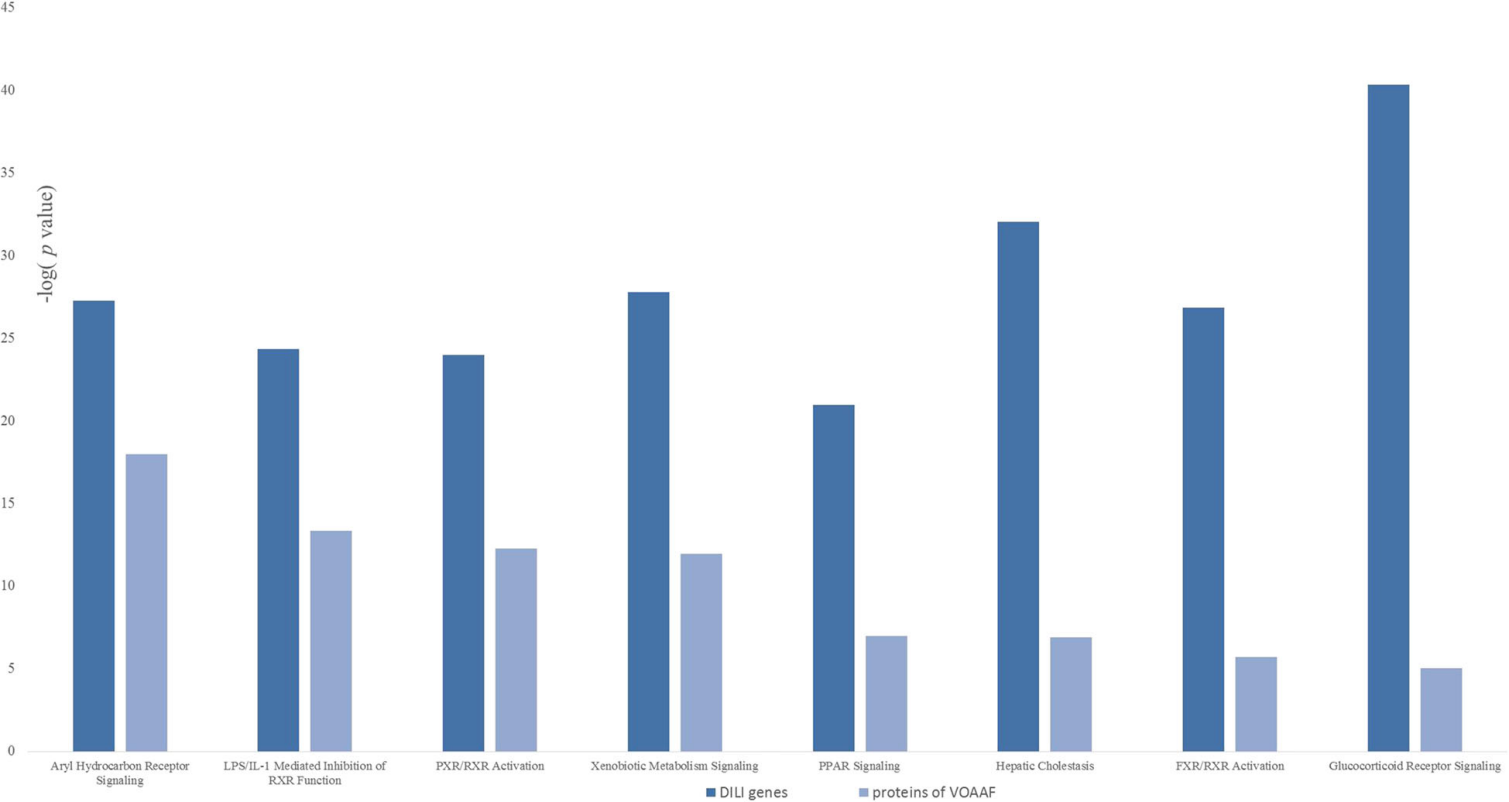
The common pathways both involved in DILI genes and target proteins of VOAAF

Canonical pathway analysis identified the pathways most significant to the data set, based on two parameters: (1) a ratio of the number of proteins from the data set that map to the pathway divided by the total number of proteins that map to the canonical pathway and (2) a *P* value calculated with Fisher’s exact test determining the probability that the association between the proteins in the dataset and the canonical pathway is explained by chance alone. To elucidate the mechanism, we chose the pathways with molecules that were both with large sum in target proteins of VOAAF and DILI genes (Fig. 5). The core analysis of the canonical pathway indicated that target proteins of VOAAF and DILI genes both involved in the pathways of aryl hydrocarbon receptor (AHR) signaling, lipopolysaccharide (LPS)/interleukin 1 (IL-1) mediated inhibition of retinoid X receptor (RXR) function, pregnane X receptor (PXR)/RXR activation, xenobiotic metabolism signaling, peroxisome proliferator-activated receptor (PPAR) signaling, hepatic cholestasis, farnesoid X receptor (FXR)/RXR activation, and glucocorticoid receptor signaling. Those pathways are all with high ratio and *P* value according to the test.

**Fig. 6:**
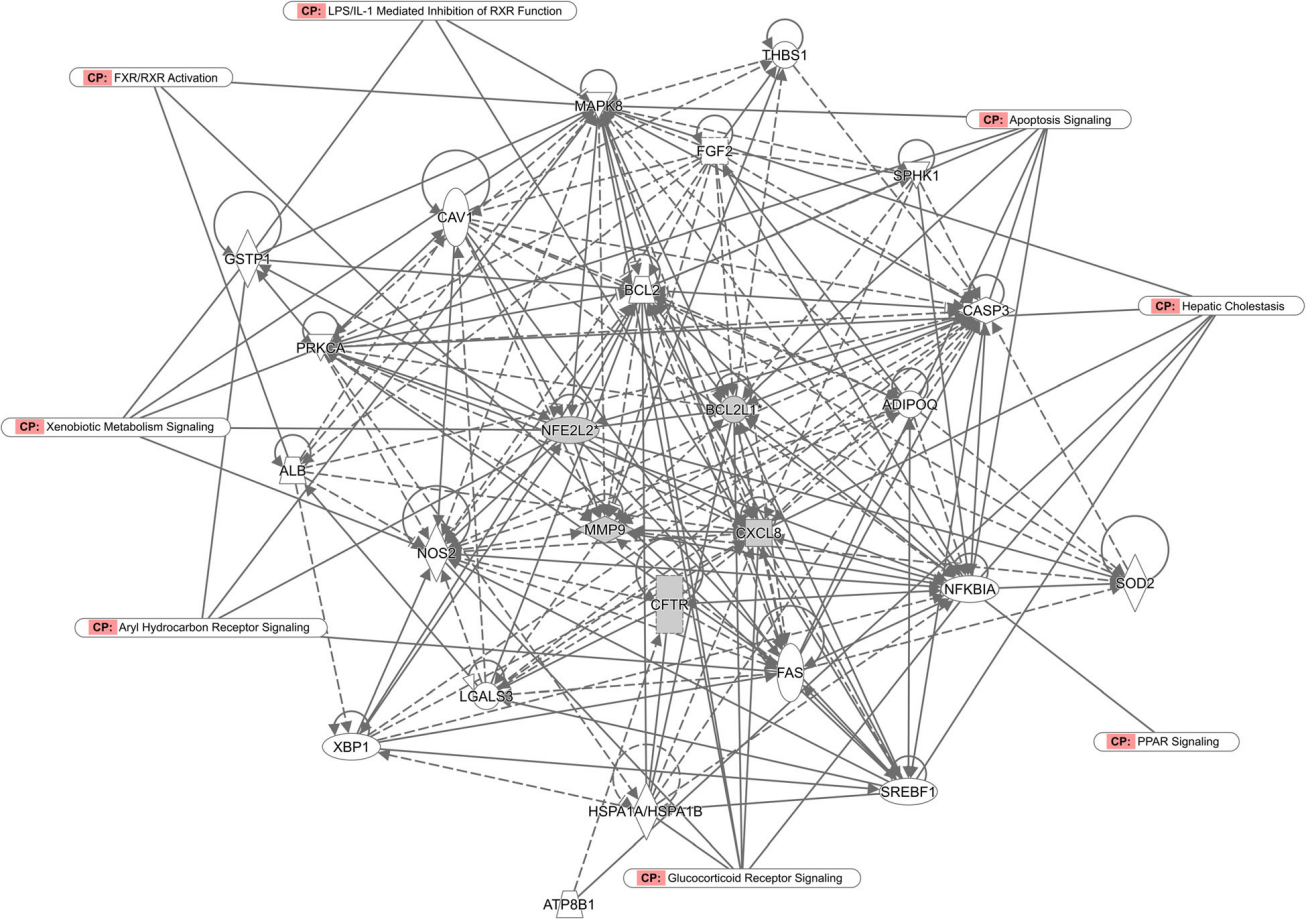
The molecular network of both DILI and VOAAF

With the comparison analysis (Fig. 6), it was found that the DILI and VOAAF networks had common pathways in gene expression, lipid metabolism, molecular transport, small molecule biochemistry. The aryl hydrocarbon receptor signaling, LPS/IL-1 mediated inhibition of RXR function were also involved.

## Discussion

According to network biology, life is a complex network which is interacted with various molecules. The occurrence and development of disease relates to a series of interacting genes or proteins, and the disease phenotype reflects the different pathological biological processes in a complex network of interactions [19]. Chinese herbal drugs have many compositions and various pharmacological effects. Bioinformatics, based on the analysis of molecular network, could reveal the biological significance of the data contained through acquiring, processing, storage, retrieval and analysis the biological experimental data [20, 21]. Based on the systems biology data, the advanced bioinformatics analysis technology has become an important means to explore mechanism and pharmacological effect of Chinese herbal drugs [22]. We hope that through analysis of molecular network this research can provide a basis for a better understanding of the molecular mechanisms of the liver injury induced by VOAAF.

The Liver Disease Group of the Chinese Medical Association defined the liver injury caused by drug hepatotoxicity as DILI [23], which includes the liver injury caused by the drug itself and/or its metabolites in the process of drug use or liver injury caused by drug allergy reaction [24], collectively called drug-induced hepatitis [25]. The onset of DILI is a complex process, and its pathogenesis has become a research hotspot in recent years. The pathogenesis of DILI in recent years is mainly considered as follows: the intrinsic hepatotoxicity mechanism, the immune idiosyncratic hepatotoxicity mechanism, the metabolic idiosyncratic hepatotoxicity mechanism, the mitochondrial damage mechanism, the biliary injury mechanism, signal transduction and liver cell death mechanism, the lipid peroxidation damage mechanism, the calcium balance and cell membrane damage mechanism [26–28]. The pathogenesis of DILI often involves toxic drugs or toxic metabolites, which causes immune reactions, or directly affects hepatocytes, eventually leading to clinical sign of hepatitis. Through IPA, it was found that the target proteins of VOAAF mainly involved the diseases and biological functions of inflammatory response, gene expression, organismal injury and abnormalities. Those are right consistent with the increase of alanine transaminase (ALT) and aspartate aminotransferase (AST) levels and the histopathologic changes of liver tissues, which have been well verified by researcher with animal experiments through oral administration of VOAAF to rats or mice [29–32].

The core analysis and the comparison analysis with the IPA software to the canonical pathway indicated that the target proteins of VOAAF and DILI genes both were involved with pathways of AHR signaling and LPS/IL-1 mediated inhibition of RXR function.

The AHR signaling is a nuclear receptor signaling, and it is mostly related with apoptosis, cell cycle regulation and xenobiotic metabolism. AHR is a ligand-activated transcription factor which locates in the cell cytoplasm, and it belongs to the superfamily of basic helix-loop-helix per-arnt-sim homology domain protein subunits. AHR is activated by its related ligand after binding with it, and thereupon it translocates into cell nucleus to formulate the dimer complex which could bind with the corresponding deoxyribonucleic acid (DNA) sequence and enhances the expression of the cytochrome P450 (CYP450) with the AHR nuclear translocator [33]. Cytochrome P1A1 (CYP1A1) and cytochromeP1A2 (CYP1A2) could metabolise many compounds into toxic substance, such as the reactive oxygen species (ROS). Through this way, CYP1A1 and CYP1A2 could influence on the mitochondria, and lead to the decrease of the mitochondrial membrane potential [34, 35]. The classic agonist of AHR, 2, 3, 7, 8-tetrachlorodibenzo-p-dioxin (TCDD) could produce extra H_2_O_2_ in the primary hepatocytes of rats and mice. TCDD could down-regulate the superoxide dismutase (SOD), catalase (CAT), glutathione peroxidase (GSH-Px) in the primary rats hepatocytes and decrease the activity of the mitochondria complex as well [34, 36]. AHR could bind DNA directly in the mitochondria of liver cells, to influence the expression of the mitochondrial proteome, and then lead to the change of the function of the mitochondria and induce the liver injury [37]. In our previous study, it was found that after the gavage of a certain amount of VOAAF to mice, a large amount of ROS would be generated, and the content of the malondialdehyde (MDA), oxidized glutathione (GSSG) in the liver tissues both increased obviously, while the antioxidation activity of the SOD and the content of the glutathione (GSH) decreased obviously [29, 32]. The mitochondria became swollen, and mitochondrial membrane potential decreased as well as the activity of Na^+^-K^+^-ATPase, Ca^2+^-ATPase and Ca^2+^-Mg^2+^-ATPase [38]. According to those results, it is proposed that the AHR signaling pathway could be the major one related to VOAAF-induced liver injury, and it mainly involves mitochondrial damage.

LPS/IL-1 mediated inhibition of RXR function is a nuclear receptor signaling. The top functions and diseases of it are lipid metabolism; molecular transport; small molecule biochemistry. Lipopolysaccharide (LPS), a major component of the outer membrane of gram-negative bacteria, potently stimulates host innate immune response. LPS binds the cysteine desulfhydrase 14 (CD14)/trithorax-like 4 (TRL4)/myeloid differentiation protein-2 (MD2) receptor complex, which promotes the secretion of pro-inflammatory cytokines like IL-1, tumor necrosis factor-α (TNF-α) in various cells, especially in macrophages. Infection, inflammation and injury down-regulate the expression of hepatic genes involved in a variety of physiological processes, collectively known as the negative hepatic acute phase response (APR). Many of the repressed genes during APR are regulated by the nuclear hormone receptor retinoid X receptor α (RXRα). In response to pro-inflammatory cytokines signaling, RXRα undergoes a c-Jun N-terminal Kinase (JNK) mediated, chromosome region maintenance 1 (CRM-1)-dependent nuclear export, leading to decreased nuclear RXRα levels and reduced nuclear DNA binding and transcriptional activity. Reduction in the expression of hepatic transport proteins (ATP-binding cassette (ABC), organic anion transporting polypeptide (OATP), multidrug resistance gene 1 (MDR1), Na^+^/taurocholate cotransporting polypeptide (NTCP)), metabolizing enzymes (CYP, glutathione transferase (GST), UDP-glycosyltransferase (UGT), SOD) and biosynthesis enzymes (CYP7A1), leads to impaired metabolism, transport and/or biosynthesis of lipid, cholesterol, bile acid and xenobiotics. A research indicated that the JNK pathway was closely related to the hepatic cells death [39]. Usually the activation of this pathway could be inhibited by the survival gene of nuclear factor kappa β (NF-κβ), so that the inhibition of the activation is transitory and non-toxic. However, the consistent activation of the pathway may lead to the cell death. The JNK pathway could be activated by many stressors, such as the ROS, ultraviolet (uv) light, receptor-interacting proteins [40], which lead to the cell death finally. Besides, the mitochondria is the major target of the JNK pathway. The consistent activation of JNK signaling pathway occurs in the mitochondria, affects the function of mitochondria and magnifies the oxidative stress, eventually leading to mitochondrial ailure and cell death. Therefore, the LPS/IL-1 mediated inhibition of RXR function involves in the signal transduction and liver cell death of DILI, indicating that the LPS/IL-1 mediated inhibition of RXR function may involve in the VOAAF-induced liver injury.

## Conclusions

In this work, 2 molecular networks and their functions were analyzed through IPA. One network identified genes related to DILI, and the other identified VOAAF target proteins. Although the VOAAF could affect various DILI-related functions and pathways in various ways, the major affected functions and pathways were analyzed in this paper, and the effects on AHR signaling and LPS/IL-1 mediated inhibition of RXR function might be crucial for elucidating the molecular mechanism of VOAAF. In future work, experiments in vitro and in vivo will be performed to verify the results by IPA.

## Additional files

Additional file 1: Table S1. DILI-related genes searched in PubChem. In the GenBank database 338 genes were identified involved in DILI. (DOCX 53 kb)

Additional file 2: Table S2. Target proteins of VOAAF searched in PubChem. Searched in the PubChem database, information on the drug targets of the VOAAF was listed. (DOCX 33 kb)

## Abbreviations

ABC: ATP-binding cassette
AHR: Aryl hydrocarbon receptor
ALT: Alanine transaminase
APR: Acute phase response
AST: Aspartate aminotransferase
CAT: Catalase
CD14: Cysteine desulfhydrase 14
CNKI: China National Knowledge Infrastructure
CRM-1: Chromosome region maintenance 1
CYP1A1: Cytochrome P1A1
CYP1A2: cytochromep1a2
CYP450: Cytochrome P450
DILI: Drug-induced liver injury
DNA: Deoxyribonucleic acid
FXR: Farnesoid X receptor
GI: Gen Info Identifier
GSH: Glutathione
GSH-Px: Glutathione peroxidase
GSSG: Oxidized glutathione
GST: Glutathione transferase
IPA: Ingenuity Pathway Analysis
JNK: Jun N-terminal Kinase
LPS: Lipopolysaccharide
MD2: Myeloid differentiation protein-2
MDA: Malondialdehyde
MDR1: Multidrug resistance gene 1
NF-κβ: Nuclear factor kappa β
NTCP: Na^+^/taurocholate cotransporting polypeptide
OATP: Organic anion transporting polypeptide
PPAR: Peroxisome proliferator-activated receptor
PPI: Protein-protein interaction
PXR: Pregnane X receptor
RXR: Retinoid X receptor
RXRα: Retinoid X receptor α
siRNAs: Small interfering RNAs
SOD: Superoxide dismutase
TCDD: 2, 3, 7, 8-tetrachlorodibenzo-p-dioxin
TCM: Traditional Chinese medicine
TNF-α: Tumor necrosis factor-α
TRL4: Trithorax-like 4
UGT: UDP-glycosyltransferase
uv: Ultraviolet
VOAAF: Volatile oils from Artemisiae Argyi Folium
