# Supplementary material for: Molecular network-based analysis of the mechanism of liver injury induced by volatile oils from Artemisiae argyi folium
Source: BMC Complement Altern Med. 2017 Nov 16;17:491. doi: 10.1186/s12906-017-1997-4 (PMC5691807; doi:10.1186/s12906-017-1997-4)
Supplement: Supplementary file 1 — DILI-related genes searched in PubChem. In the GenBank database 338 genes were identified involved in DILI. (DOCX 53 kb) [file 12906_2017_1997_MOESM1_ESM.docx]

**Table S1.** DILI-related genes searched in PubChem

| **No.** | **Target proteins** | **GI number** | **Descriptions** | **Species** |
| --- | --- | --- | --- | --- |
| 1 | HLA-B | 3106 | major histocompatibility complex, class I, B | [Homo sapiens (human)] |
| 2 | STAT3 | 6774 | signal transducer and activator of transcription 3 | [Homo sapiens (human)] |
| 3 | HLA-DQB1 | 3119 | major histocompatibility complex, class II, DQ beta 1 | [Homo sapiens (human)] |
| 4 | IL10 | 3586 | interleukin 10 | [Homo sapiens (human)] |
| 5 | IL6 | 3569 | interleukin 6 | [Homo sapiens (human)] |
| 6 | CYP2E1 | 1571 | cytochrome P450 family 2 subfamily E member 1 | [Homo sapiens (human)] |
| 7 | CTLA4 | 1493 | cytotoxic T-lymphocyte associated protein 4 | [Homo sapiens (human)] |
| 8 | TP53 | 7157 | tumor protein p53 | [Homo sapiens (human)] |
| 9 | CRP | 1401 | C-reactive protein, pentraxin-related | [Homo sapiens (human)] |
| 10 | GSTM1 | 2944 | glutathione S-transferase mu 1 | [Homo sapiens (human)] |
| 11 | CYP2C19 | 1557 | cytochrome P450 family 2 subfamily C member 19 | [Homo sapiens (human)] |
| 12 | CXCL10 | 3627 | C-X-C motif chemokine ligand 10 | [Homo sapiens (human)] |
| 13 | IL2RA | 3559 | interleukin 2 receptor subunit alpha | [Homo sapiens (human)] |
| 14 | GSTP1 | 2950 | glutathione S-transferase pi 1 | [Homo sapiens (human)] |
| 15 | CYP2C9 | 1559 | cytochrome P450 family 2 subfamily C member 9 | [Homo sapiens (human)] |
| 16 | CYP3A4 | 1576 | cytochrome P450 family 3 subfamily A member 4 | [Homo sapiens (human)] |
| 17 | UGT1A1 | 54658 | UDP glucuronosyltransferase family 1 member A1 | [Homo sapiens (human)] |
| 18 | NLRP3 | 114548 | NLR family, pyrin domain containing 3 | [Homo sapiens (human)] |
| 19 | TNFSF11 | 8600 | tumor necrosis factor superfamily member 11 | [Homo sapiens (human)] |
| 20 | MCTP2 | 55784 | multiple C2 domains, transmembrane 2 | [Homo sapiens (human)] |
| 21 | PPARG | 5468 | peroxisome proliferator activated receptor gamma | [Homo sapiens (human)] |
| 22 | NAT2 | 10 | N-acetyltransferase 2 (arylamine N-acetyltransferase) | [Homo sapiens (human)] |
| 23 | ABCB1 | 5243 | ATP binding cassette subfamily B member 1 | [Homo sapiens (human)] |
| 24 | CXCL8 | 3576 | C-X-C motif chemokine ligand 8 | [Homo sapiens (human)] |
| 25 | CTNNB1 | 1499 | catenin beta 1 | [Homo sapiens (human)] |
| 26 | SERPINE1 | 5054 | serpin family E member 1 | [Homo sapiens (human)] |
| 27 | HLA-A | 3105 | major histocompatibility complex, class I, A | [Homo sapiens (human)] |
| 28 | AKT1 | 207 | v-akt murine thymoma viral oncogene homolog 1 | [Homo sapiens (human)] |
| 29 | ABCB11 | 8647 | ATP binding cassette subfamily B member 11 | [Homo sapiens (human)] |
| 30 | HLA-DRB1 | 3123 | major histocompatibility complex, class II, DR beta 1 | [Homo sapiens (human)] |
| 31 | IL4 | 3565 | interleukin 4 | [Homo sapiens (human)] |
| 32 | BIRC5 | 332 | baculoviral IAP repeat containing 5 | [Homo sapiens (human)] |
| 33 | FABP1 | 2168 | fatty acid binding protein 1 | [Homo sapiens (human)] |
| 34 | MIR122 | 406906 | microRNA 122 | [Homo sapiens (human)] |
| 35 | SQSTM1 | 8878 | sequestosome 1 | [Homo sapiens (human)] |
| 36 | SOCS3 | 9021 | suppressor of cytokine signaling 3 | [Homo sapiens (human)] |
| 37 | CYP1A1 | 1543 | cytochrome P450 family 1 subfamily A member 1 | [Homo sapiens (human)] |
| 38 | NOTCH1 | 4851 | notch 1 | [Homo sapiens (human)] |
| 39 | MET | 4233 | MET proto-oncogene, receptor tyrosine kinase | [Homo sapiens (human)] |
| 40 | NPPB | 4879 | natriuretic peptide B | [Homo sapiens (human)] |
| 41 | ARG1 | 383 | arginase 1 | [Homo sapiens (human)] |
| 42 | HSPA1A | 3303 | heat shock protein family A (Hsp70) member 1A | [Homo sapiens (human)] |
| 43 | ALG10B | 144245 | ALG10B, alpha-1,2-glucosyltransferase | [Homo sapiens (human)] |
| 44 | NFE2L2 | 4780 | nuclear factor, erythroid 2 like 2 | [Homo sapiens (human)] |
| 45 | CDKN1A | 1026 | cyclin-dependent kinase inhibitor 1A | [Homo sapiens (human)] |
| 46 | GDF15 | 9518 | growth differentiation factor 15 | [Homo sapiens (human)] |
| 47 | KRT18 | 3875 | keratin 18 | [Homo sapiens (human)] |
| 48 | FAS | 355 | Fas cell surface death receptor | [Homo sapiens (human)] |
| 49 | NR1I2 | 8856 | nuclear receptor subfamily 1 group I member 2 | [Homo sapiens (human)] |
| 50 | COMT | 1312 | catechol-O-methyltransferase | [Homo sapiens (human)] |
| 51 | HIF1A | 3091 | hypoxia inducible factor 1 alpha subunit | [Homo sapiens (human)] |
| 52 | PTGS2 | 5743 | prostaglandin-endoperoxide synthase 2 | [Homo sapiens (human)] |
| 53 | MMP9 | 4318 | matrix metallopeptidase 9 | [Homo sapiens (human)] |
| 54 | SIRT1 | 23411 | sirtuin 1 | [Homo sapiens (human)] |
| 55 | CD40 | 958 | CD40 molecule | [Homo sapiens (human)] |
| 56 | MMP2 | 4313 | matrix metallopeptidase 2 | [Homo sapiens (human)] |
| 57 | ADIPOQ | 9370 | adiponectin, C1Q and collagen domain containing | [Homo sapiens (human)] |
| 58 | ABCC2 | 1244 | ATP binding cassette subfamily C member 2 | [Homo sapiens (human)] |
| 59 | ANGPT2 | 285 | angiopoietin 2 | [Homo sapiens (human)] |
| 60 | CASP8 | 841 | caspase 8 | [Homo sapiens (human)] |
| 61 | ATP8B1 | 5205 | ATPase phospholipid transporting 8B1 | [Homo sapiens (human)] |
| 62 | VEGFA | 7422 | vascular endothelial growth factor A | [Homo sapiens (human)] |
| 63 | IFNG | 3458 | interferon, gamma | [Homo sapiens (human)] |
| 64 | KRT8 | 3856 | keratin 8 | [Homo sapiens (human)] |
| 65 | CCL2 | 6347 | C-C motif chemokine ligand 2 | [Homo sapiens (human)] |
| 66 | IL1B | 3553 | interleukin 1 beta | [Homo sapiens (human)] |
| 67 | EGFR | 1956 | epidermal growth factor receptor | [Homo sapiens (human)] |
| 68 | BCL2 | 596 | B-cell CLL/lymphoma 2 | [Homo sapiens (human)] |
| 69 | AGER | 177 | advanced glycosylation end product-specific receptor | [Homo sapiens (human)] |
| 70 | ABCB4 | 5244 | ATP binding cassette subfamily B member 4 | [Homo sapiens (human)] |
| 71 | HMOX1 | 3162 | heme oxygenase 1 | [Homo sapiens (human)] |
| 72 | ICAM1 | 3383 | intercellular adhesion molecule 1 | [Homo sapiens (human)] |
| 73 | MIR21 | 406991 | microRNA 21 | [Homo sapiens (human)] |
| 74 | PTEN | 5728 | phosphatase and tensin homolog | [Homo sapiens (human)] |
| 75 | EPO | 2056 | erythropoietin | [Homo sapiens (human)] |
| 76 | PPARA | 5465 | peroxisome proliferator activated receptor alpha | [Homo sapiens (human)] |
| 77 | CD40LG | 959 | CD40 ligand | [Homo sapiens (human)] |
| 78 | ERBB2 | 2064 | erb-b2 receptor tyrosine kinase 2 | [Homo sapiens (human)] |
| 79 | NFKB1 | 4790 | nuclear factor of kappa light polypeptide gene enhancer in B-cells 1 | [Homo sapiens (human)] |
| 80 | E2F1 | 1869 | E2F transcription factor 1 | [Homo sapiens (human)] |
| 81 | GPX1 | 2876 | glutathione peroxidase 1 | [Homo sapiens (human)] |
| 82 | NOS2 | 4843 | nitric oxide synthase 2 | [Homo sapiens (human)] |
| 83 | CYP1A2 | 1544 | cytochrome P450 family 1 subfamily A member 2 | [Homo sapiens (human)] |
| 84 | EGF | 1950 | epidermal growth factor | [Homo sapiens (human)] |
| 85 | CD44 | 960 | CD44 molecule (Indian blood group) [Homo sapiens (human)] | [Homo sapiens (human)] |
| 86 | CD14 | 929 | CD14 molecule | [Homo sapiens (human)] |
| 87 | ADRB2 | 154 | adrenoceptor beta 2 | [Homo sapiens (human)] |
| 88 | APOA1 | 335 | apolipoprotein A-I | [Homo sapiens (human)] |
| 89 | HGF | 3082 | hepatocyte growth factor | [Homo sapiens (human)] |
| 90 | MTOR | 2475 | mechanistic target of rapamycin | [Homo sapiens (human)] |
| 91 | PDCD1 | 5133 | programmed cell death 1 | [Homo sapiens (human)] |
| 92 | PRKCZ | 5590 | protein kinase C zeta | [Homo sapiens (human)] |
| 93 | PLAUR | 5329 | plasminogen activator, urokinase receptor | [Homo sapiens (human)] |
| 94 | HMGB1 | 3146 | high mobility group box 1 | [Homo sapiens (human)] |
| 95 | EDN1 | 1906 | endothelin 1 | [Homo sapiens (human)] |
| 96 | CREBBP | 1387 | CREB binding protein | [Homo sapiens (human)] |
| 97 | SERPINA1 | 5265 | serpin family A member 1 | [Homo sapiens (human)] |
| 98 | RETN | 56729 | resistin | [Homo sapiens (human)] |
| 99 | F3 | 2152 | coagulation factor III, tissue factor | [Homo sapiens (human)] |
| 100 | GSK3B | 2932 | glycogen synthase kinase 3 beta | [Homo sapiens (human)] |
| 101 | MAPK1 | 5594 | mitogen-activated protein kinase 1 | [Homo sapiens (human)] |
| 102 | FGF21 | 26291 | fibroblast growth factor 21 | [Homo sapiens (human)] |
| 103 | ACE | 1636 | angiotensin I converting enzyme | [Homo sapiens (human)] |
| 104 | FLT1 | 2321 | fms related tyrosine kinase 1 | [Homo sapiens (human)] |
| 105 | ALB | 213 | albumin | [Homo sapiens (human)] |
| 106 | BRAF | 673 | B-Raf proto-oncogene, serine/threonine kinase | [Homo sapiens (human)] |
| 107 | IL17A | 3605 | interleukin 17A | [Homo sapiens (human)] |
| 108 | ITGB1 | 3688 | integrin subunit beta 1 | [Homo sapiens (human)] |
| 109 | HSPB1 | 3315 | heat shock protein family B (small) member 1 | [Homo sapiens (human)] |
| 110 | CYP3A5 | 1577 | cytochrome P450 family 3 subfamily A member 5 | [Homo sapiens (human)] |
| 111 | AICDA | 57379 | activation-induced cytidine deaminase | [Homo sapiens (human)] |
| 112 | CCND1 | 595 | cyclin D1 | [Homo sapiens (human)] |
| 113 | IL1A | 3552 | interleukin 1 alpha | [Homo sapiens (human)] |
| 114 | TNFSF10 | 8743 | tumor necrosis factor superfamily member 10 | [Homo sapiens (human)] |
| 115 | ATR | 545 | ATR serine/threonine kinase | [Homo sapiens (human)] |
| 116 | ABCC1 | 4363 | ATP binding cassette subfamily C member 1 | [Homo sapiens (human)] |
| 117 | TLR3 | 7098 | toll like receptor 3 | [Homo sapiens (human)] |
| 118 | NOS3 | 4846 | nitric oxide synthase 3 | [Homo sapiens (human)] |
| 119 | MCL1 | 4170 | myeloid cell leukemia 1 | [Homo sapiens (human)] |
| 120 | MAPK3 | 5595 | mitogen-activated protein kinase 3 | [Homo sapiens (human)] |
| 121 | IL1RN | 3557 | interleukin 1 receptor antagonist | [Homo sapiens (human)] |
| 122 | TIMP2 | 7077 | TIMP metallopeptidase inhibitor 2 | [Homo sapiens (human)] |
| 123 | SMAD3 | 4088 | SMAD family member 3 | [Homo sapiens (human)] |
| 124 | MMP1 | 4312 | matrix metallopeptidase 1 | [Homo sapiens (human)] |
| 125 | BAX | 581 | BCL2-associated X protein | [Homo sapiens (human)] |
| 126 | VDR | 7421 | vitamin D (1,25- dihydroxyvitamin D3) receptor | [Homo sapiens (human)] |
| 127 | SHH | 6469 | sonic hedgehog | [Homo sapiens (human)] |
| 128 | LGALS1 | 3956 | lectin, galactoside binding soluble 1 | [Homo sapiens (human)] |
| 129 | FGF2 | 2247 | fibroblast growth factor 2 | [Homo sapiens (human)] |
| 130 | MIR155 | 406947 | microRNA 155 | [Homo sapiens (human)] |
| 131 | MAPK8 | 5599 | mitogen-activated protein kinase 8 | [Homo sapiens (human)] |
| 132 | TGFB2 | 7042 | transforming growth factor beta 2 | [Homo sapiens (human)] |
| 133 | BSG | 682 | basigin (Ok blood group) | [Homo sapiens (human)] |
| 134 | ADAM17 | 6868 | ADAM metallopeptidase domain 17 | [Homo sapiens (human)] |
| 135 | THBS1 | 7057 | thrombospondin 1 | [Homo sapiens (human)] |
| 136 | IL37 | 27178 | interleukin 37 | [Homo sapiens (human)] |
| 137 | COL1A1 | 1277 | collagen type I alpha 1 | [Homo sapiens (human)] |
| 138 | S100B | 6285 | S100 calcium binding protein B | [Homo sapiens (human)] |
| 139 | BECN1 | 8678 | beclin 1, autophagy related | [Homo sapiens (human)] |
| 140 | SHC1 | 6464 | SHC (Src homology 2 domain containing) transforming protein 1 | [Homo sapiens (human)] |
| 141 | ABCC4 | 10257 | ATP binding cassette subfamily C member 4 | [Homo sapiens (human)] |
| 142 | CX3CR1 | 1524 | C-X3-C motif chemokine receptor 1 | [Homo sapiens (human)] |
| 143 | LGALS3 | 3958 | lectin, galactoside binding soluble 3 | [Homo sapiens (human)] |
| 144 | IGF1R | 3480 | insulin like growth factor 1 receptor | [Homo sapiens (human)] |
| 145 | CD34 | 947 | CD34 molecule | [Homo sapiens (human)] |
| 146 | MMP7 | 4316 | matrix metallopeptidase 7 | [Homo sapiens (human)] |
| 147 | IL13 | 3596 | interleukin 13 | [Homo sapiens (human)] |
| 148 | ABCC3 | 8714 | ATP binding cassette subfamily C member 3 | [Homo sapiens (human)] |
| 149 | SLC9A1 | 6548 | solute carrier family 9 member A1 | [Homo sapiens (human)] |
| 150 | XBP1 | 7494 | X-box binding protein 1 | [Homo sapiens (human)] |
| 151 | UCP2 | 7351 | uncoupling protein 2 (mitochondrial, proton carrier) | [Homo sapiens (human)] |
| 152 | MIR146A | 406938 | microRNA 146a | [Homo sapiens (human)] |
| 153 | MIR210 | 406992 | microRNA 210 | [Homo sapiens (human)] |
| 154 | RHOA | 387 | ras homolog family member A | [Homo sapiens (human)] |
| 155 | SGK1 | 6446 | serum/glucocorticoid regulated kinase 1 | [Homo sapiens (human)] |
| 156 | CXCR2 | 3579 | C-X-C motif chemokine receptor 2 | [Homo sapiens (human)] |
| 157 | BMP7 | 655 | bone morphogenetic protein 7 | [Homo sapiens (human)] |
| 158 | PRKCA | 5578 | protein kinase C alpha | [Homo sapiens (human)] |
| 159 | MMP12 | 4321 | matrix metallopeptidase 12 | [Homo sapiens (human)] |
| 160 | HSPB2 | 3316 | heat shock protein family B (small) member 2 | [Homo sapiens (human)] |
| 161 | MAP2K1 | 5604 | mitogen-activated protein kinase kinase 1 | [Homo sapiens (human)] |
| 162 | HDAC3 | 8841 | histone deacetylase 3 | [Homo sapiens (human)] |
| 163 | SPHK1 | 8877 | sphingosine kinase 1 | [Homo sapiens (human)] |
| 164 | POSTN | 10631 | periostin, osteoblast specific factor | [Homo sapiens (human)] |
| 165 | CPB2 | 1361 | carboxypeptidase B2 | [Homo sapiens (human)] |
| 166 | VHL | 7428 | von Hippel-Lindau tumor suppressor | [Homo sapiens (human)] |
| 167 | CES1 | 1066 | carboxylesterase 1 | [Homo sapiens (human)] |
| 168 | B2M | 567 | beta-2-microglobulin | [Homo sapiens (human)] |
| 169 | IL21 | 59067 | interleukin 21 | [Homo sapiens (human)] |
| 170 | NFKBIA | 4792 | NFKB inhibitor alpha | [Homo sapiens (human)] |
| 171 | DKK1 | 22943 | dickkopf WNT signaling pathway inhibitor 1 | [Homo sapiens (human)] |
| 172 | OCLN | 1E+08 | occludin | [Homo sapiens (human)] |
| 173 | FABP3 | 2170 | fatty acid binding protein 3 | [Homo sapiens (human)] |
| 174 | MT2A | 4502 | metallothionein 2A | [Homo sapiens (human)] |
| 175 | UGT1A6 | 54578 | UDP glucuronosyltransferase family 1 member A6 | [Homo sapiens (human)] |
| 176 | CYP2C8 | 1558 | cytochrome P450 family 2 subfamily C member 8 | [Homo sapiens (human)] |
| 177 | HMGCR | 3156 | 3-hydroxy-3-methylglutaryl-CoA reductase | [Homo sapiens (human)] |
| 178 | PRDX2 | 7001 | peroxiredoxin 2 | [Homo sapiens (human)] |
| 179 | COL18A1 | 80781 | collagen type XVIII alpha 1 | [Homo sapiens (human)] |
| 180 | SLC29A1 | 2030 | solute carrier family 29 member 1 (Augustine blood group) | [Homo sapiens (human)] |
| 181 | C4B | 721 | complement component 4B (Chido blood group) | [Homo sapiens (human)] |
| 182 | IDH2 | 3418 | isocitrate dehydrogenase 2 (NADP+), mitochondrial | [Homo sapiens (human)] |
| 183 | YAP1 | 10413 | Yes associated protein 1 | [Homo sapiens (human)] |
| 184 | SLPI | 6590 | secretory leukocyte peptidase inhibitor | [Homo sapiens (human)] |
| 185 | TERT | 7015 | telomerase reverse transcriptase | [Homo sapiens (human)] |
| 186 | SREBF1 | 6720 | sterol regulatory element binding transcription factor 1 | [Homo sapiens (human)] |
| 187 | PPARD | 5467 | peroxisome proliferator activated receptor delta | [Homo sapiens (human)] |
| 188 | TGFBR3 | 7049 | transforming growth factor beta receptor III | [Homo sapiens (human)] |
| 189 | JUNB | 3726 | jun B proto-oncogene | [Homo sapiens (human)] |
| 190 | NDRG2 | 57447 | NDRG family member 2 | [Homo sapiens (human)] |
| 191 | PRKAA2 | 5563 | protein kinase AMP-activated catalytic subunit alpha 2 | [Homo sapiens (human)] |
| 192 | IFNL4 | 1E+08 | interferon, lambda 4 (gene/pseudogene) | [Homo sapiens (human)] |
| 193 | IFNL3 | 282617 | interferon, lambda 3 | [Homo sapiens (human)] |
| 194 | IRS1 | 3667 | insulin receptor substrate 1 | [Homo sapiens (human)] |
| 195 | MAPK9 | 5601 | mitogen-activated protein kinase 9 | [Homo sapiens (human)] |
| 196 | BBC3 | 27113 | BCL2 binding component 3 | [Homo sapiens (human)] |
| 197 | OGG1 | 4968 | 8-oxoguanine DNA glycosylase | [Homo sapiens (human)] |
| 198 | PTK2 | 5747 | protein tyrosine kinase 2 | [Homo sapiens (human)] |
| 199 | CNR1 | 1268 | cannabinoid receptor 1 (brain) | [Homo sapiens (human)] |
| 200 | PTN | 5764 | pleiotrophin | [Homo sapiens (human)] |
| 201 | GAL | 51083 | galanin/GMAP prepropeptide | [Homo sapiens (human)] |
| 202 | AQP1 | 358 | aquaporin 1 (Colton blood group) | [Homo sapiens (human)] |
| 203 | PPARGC1A | 10891 | PPARG coactivator 1 alpha | [Homo sapiens (human)] |
| 204 | ELAVL1 | 1994 | ELAV like RNA binding protein 1 | [Homo sapiens (human)] |
| 205 | IGF2 | 3481 | insulin like growth factor 2 | [Homo sapiens (human)] |
| 206 | RAC1 | 5879 | ras-related C3 botulinum toxin substrate 1 (rho family, small GTP binding protein Rac1) | [Homo sapiens (human)] |
| 207 | ITGA6 | 3655 | integrin subunit alpha 6 | [Homo sapiens (human)] |
| 208 | APLN | 8862 | apelin | [Homo sapiens (human)] |
| 209 | CYP7A1 | 1581 | cytochrome P450 family 7 subfamily A member 1 | [Homo sapiens (human)] |
| 210 | GCG | 2641 | glucagon | [Homo sapiens (human)] |
| 211 | BMP6 | 654 | bone morphogenetic protein 6 | [Homo sapiens (human)] |
| 212 | CYP19A1 | 1588 | cytochrome P450 family 19 subfamily A member 1 | [Homo sapiens (human)] |
| 213 | ADAM12 | 8038 | ADAM metallopeptidase domain 12 | [Homo sapiens (human)] |
| 214 | BAG3 | 9531 | BCL2 associated athanogene 3 | [Homo sapiens (human)] |
| 215 | ATP7B | 540 | ATPase copper transporting beta | [Homo sapiens (human)] |
| 216 | LOX | 4015 | lysyl oxidase | [Homo sapiens (human)] |
| 217 | TFEB | 7942 | transcription factor EB | [Homo sapiens (human)] |
| 218 | MDM2 | 4193 | MDM2 proto-oncogene | [Homo sapiens (human)] |
| 219 | CASP10 | 843 | caspase 10 | [Homo sapiens (human)] |
| 220 | NPY | 4852 | neuropeptide Y | [Homo sapiens (human)] |
| 221 | MIR145 | 406937 | microRNA 145 | [Homo sapiens (human)] |
| 222 | KCNQ1 | 3784 | potassium voltage-gated channel subfamily Q member 1 | [Homo sapiens (human)] |
| 223 | SLC1A2 | 6506 | solute carrier family 1 member 2 | [Homo sapiens (human)] |
| 224 | FABP4 | 2167 | fatty acid binding protein 4 | [Homo sapiens (human)] |
| 225 | SLC22A1 | 6580 | solute carrier family 22 member 1 | [Homo sapiens (human)] |
| 226 | UTS2 | 10911 | urotensin 2 | [Homo sapiens (human)] |
| 227 | TUBB3 | 10381 | tubulin beta 3 class III | [Homo sapiens (human)] |
| 228 | CYP2B6 | 1555 | cytochrome P450 family 2 subfamily B member 6 | [Homo sapiens (human)] |
| 229 | RORA | 6095 | RAR related orphan receptor A | [Homo sapiens (human)] |
| 230 | KCNE1 | 3753 | potassium voltage-gated channel subfamily E regulatory subunit 1 | [Homo sapiens (human)] |
| 231 | RUNX1 | 861 | runt related transcription factor 1 | [Homo sapiens (human)] |
| 232 | LIPC | 3990 | lipase C, hepatic type | [Homo sapiens (human)] |
| 233 | CARD9 | 64170 | caspase recruitment domain family member 9 | [Homo sapiens (human)] |
| 234 | BGLAP | 632 | bone gamma-carboxyglutamate protein | [Homo sapiens (human)] |
| 235 | YY1 | 7528 | YY1 transcription factor | [Homo sapiens (human)] |
| 236 | PLIN2 | 123 | perilipin 2 | [Homo sapiens (human)] |
| 237 | GCLC | 2729 | glutamate-cysteine ligase catalytic subunit | [Homo sapiens (human)] |
| 238 | PAWR | 5074 | pro-apoptotic WT1 regulator | [Homo sapiens (human)] |
| 239 | SERPINB3 | 6317 | serpin family B member 3 | [Homo sapiens (human)] |
| 240 | BIRC2 | 329 | baculoviral IAP repeat containing 2 | [Homo sapiens (human)] |
| 241 | SLCO1B1 | 10599 | solute carrier organic anion transporter family member 1B1 | [Homo sapiens (human)] |
| 242 | ST6GAL1 | 6480 | ST6 beta-galactosamide alpha-2,6-sialyltranferase 1 | [Homo sapiens (human)] |
| 243 | TNF | 7124 | tumor necrosis factor | [Homo sapiens (human)] |
| 244 | HCP5 | 10866 | HLA complex P5 (non-protein coding) | [Homo sapiens (human)] |
| 245 | MTHFR | 4524 | methylenetetrahydrofolate reductase (NAD(P)H) | [Homo sapiens (human)] |
| 246 | FASLG | 356 | Fas ligand | [Homo sapiens (human)] |
| 247 | MAPK14 | 1432 | mitogen-activated protein kinase 14 | [Homo sapiens (human)] |
| 248 | ALDH2 | 217 | aldehyde dehydrogenase 2 family (mitochondrial) | [Homo sapiens (human)] |
| 249 | TNFAIP3 | 7128 | TNF alpha induced protein 3 | [Homo sapiens (human)] |
| 250 | SOD2 | 6648 | superoxide dismutase 2, mitochondrial | [Homo sapiens (human)] |
| 251 | KDR | 3791 | kinase insert domain receptor | [Homo sapiens (human)] |
| 252 | PTX3 | 5806 | pentraxin 3 | [Homo sapiens (human)] |
| 253 | CXCL12 | 6387 | C-X-C motif chemokine ligand 12 | [Homo sapiens (human)] |
| 254 | OR5H2 | 79310 | olfactory receptor family 5 subfamily H member 2 | [Homo sapiens (human)] |
| 255 | CAV1 | 857 | caveolin 1 | [Homo sapiens (human)] |
| 256 | TGFB1 | 7040 | transforming growth factor beta 1 | [Homo sapiens (human)] |
| 257 | CEBPB | 1051 | CCAAT/enhancer binding protein beta | [Homo sapiens (human)] |
| 258 | SOD1 | 6647 | superoxide dismutase 1, soluble | [Homo sapiens (human)] |
| 259 | ADAMTS13 | 11093 | ADAM metallopeptidase with thrombospondin type 1 motif 13 | [Homo sapiens (human)] |
| 260 | LCN2 | 3934 | lipocalin 2 | [Homo sapiens (human)] |
| 261 | IL18 | 3606 | interleukin 18 | [Homo sapiens (human)] |
| 262 | NAMPT | 10135 | nicotinamide phosphoribosyltransferase | [Homo sapiens (human)] |
| 263 | JUN | 3725 | jun proto-oncogene | [Homo sapiens (human)] |
| 264 | IL2 | 3558 | interleukin 2 | [Homo sapiens (human)] |
| 265 | CASP3 | 836 | caspase 3 | [Homo sapiens (human)] |
| 266 | TNFRSF11B | 4982 | tumor necrosis factor receptor superfamily member 11b | [Homo sapiens (human)] |
| 267 | TGFBI | 7045 | transforming growth factor beta induced | [Homo sapiens (human)] |
| 268 | CXCR4 | 7852 | C-X-C motif chemokine receptor 4 | [Homo sapiens (human)] |
| 269 | XRCC1 | 7515 | X-ray repair complementing defective repair in Chinese hamster cells 1 | [Homo sapiens (human)] |
| 270 | CFTR | 1080 | cystic fibrosis transmembrane conductance regulator | [Homo sapiens (human)] |
| 271 | SELP | 6403 | selectin P | [Homo sapiens (human)] |
| 272 | RELA | 5970 | v-rel avian reticuloendotheliosis viral oncogene homolog A | [Homo sapiens (human)] |
| 273 | CDH1 | 999 | cadherin 1 | [Homo sapiens (human)] |
| 274 | PDGFB | 5155 | platelet derived growth factor subunit B | [Homo sapiens (human)] |
| 275 | CTGF | 1490 | connective tissue growth factor | [Homo sapiens (human)] |
| 276 | BMI1 | 648 | BMI1 proto-oncogene, polycomb ring finger | [Homo sapiens (human)] |
| 277 | JAK2 | 3717 | Janus kinase 2 | [Homo sapiens (human)] |
| 278 | IL15 | 3600 | interleukin 15 | [Homo sapiens (human)] |
| 279 | PRDX6 | 9588 | peroxiredoxin 6 | [Homo sapiens (human)] |
| 280 | PON1 | 5444 | paraoxonase 1 | [Homo sapiens (human)] |
| 281 | NTRK1 | 4914 | neurotrophic receptor tyrosine kinase 1 | [Homo sapiens (human)] |
| 282 | CCL5 | 6352 | C-C motif chemokine ligand 5 | [Homo sapiens (human)] |
| 283 | BCL2L1 | 598 | BCL2 like 1 | [Homo sapiens (human)] |
| 284 | PROM1 | 8842 | prominin 1 | [Homo sapiens (human)] |
| 285 | FOXO3 | 2309 | forkhead box O3 | [Homo sapiens (human)] |
| 286 | SRC | 6714 | SRC proto-oncogene, non-receptor tyrosine kinase | [Homo sapiens (human)] |
| 287 | PLAU | 5328 | plasminogen activator, urokinase | [Homo sapiens (human)] |
| 288 | NEWENTRY | 192343 | Record to support submission of GeneRIFs for a gene not in Gene (human; man). | [Homo sapiens (human)] |
| 289 | TNFRSF10B | 8795 | tumor necrosis factor receptor superfamily member 10b | [Homo sapiens (human)] |
| 290 | LYN | 4067 | LYN proto-oncogene, Src family tyrosine kinase | [Homo sapiens (human)] |
| 291 | PTPN1 | 5770 | protein tyrosine phosphatase, non-receptor type 1 | [Homo sapiens (human)] |
| 292 | ITGAV | 3685 | integrin subunit alpha V | [Homo sapiens (human)] |
| 293 | CD38 | 952 | CD38 molecule | [Homo sapiens (human)] |
| 294 | S100A9 | 6280 | S100 calcium binding protein A9 | [Homo sapiens (human)] |
| 295 | PIK3CG | 5294 | phosphatidylinositol-4,5-bisphosphate 3-kinase catalytic subunit gamma | [Homo sapiens (human)] |
| 296 | ANXA2 | 302 | annexin A2 | [Homo sapiens (human)] |
| 297 | SP1 | 6667 | Sp1 transcription factor | [Homo sapiens (human)] |
| 298 | S100A8 | 6279 | S100 calcium binding protein A8 | [Homo sapiens (human)] |
| 299 | ANXA1 | 301 | annexin A1 | [Homo sapiens (human)] |
| 300 | C5 | 727 | complement component 5 | [Homo sapiens (human)] |
| 301 | MPO | 4353 | myeloperoxidase | [Homo sapiens (human)] |
| 302 | CCR6 | 1235 | C-C motif chemokine receptor 6 | [Homo sapiens (human)] |
| 303 | SKP2 | 6502 | S-phase kinase-associated protein 2, E3 ubiquitin protein ligase | [Homo sapiens (human)] |
| 304 | MIR16-1 | 406950 | microRNA 16-1 | [Homo sapiens (human)] |
| 305 | MYC | 4609 | v-myc avian myelocytomatosis viral oncogene homolog | [Homo sapiens (human)] |
| 306 | ADA | 100 | adenosine deaminase | [Homo sapiens (human)] |
| 307 | TXN | 7295 | thioredoxin | [Homo sapiens (human)] |
| 308 | CLU | 1191 | clusterin | [Homo sapiens (human)] |
| 309 | PDGFRA | 5156 | platelet derived growth factor receptor alpha | [Homo sapiens (human)] |
| 310 | STAT4 | 6775 | signal transducer and activator of transcription 4 | [Homo sapiens (human)] |
| 311 | CD276 | 80381 | CD276 molecule | [Homo sapiens (human)] |
| 312 | HLA-DQA1 | 3117 | major histocompatibility complex, class II, DQ alpha 1 | [Homo sapiens (human)] |
| 313 | ABCG2 | 9429 | ATP binding cassette subfamily G member 2 (Junior blood group) | [Homo sapiens (human)] |
| 314 | FASN | 2194 | fatty acid synthase | [Homo sapiens (human)] |
| 315 | HSPA5 | 3309 | heat shock protein family A (Hsp70) member 5 | [Homo sapiens (human)] |
| 316 | CD86 | 942 | CD86 molecule | [Homo sapiens (human)] |
| 317 | CD274 | 29126 | CD274 molecule | [Homo sapiens (human)] |
| 318 | NR1I3 | 9970 | nuclear receptor subfamily 1 group I member 3 | [Homo sapiens (human)] |
| 319 | NTS | 4922 | neurotensin | [Homo sapiens (human)] |
| 320 | PRDX1 | 5052 | peroxiredoxin 1 | [Homo sapiens (human)] |
| 321 | FOXP3 | 50943 | forkhead box P3 | [Homo sapiens (human)] |
| 322 | IGFBP2 | 3485 | insulin like growth factor binding protein 2 | [Homo sapiens (human)] |
| 323 | SMPD1 | 6609 | sphingomyelin phosphodiesterase 1 | [Homo sapiens (human)] |
| 324 | KLK3 | 354 | kallikrein related peptidase 3 | [Homo sapiens (human)] |
| 325 | NPC1L1 | 29881 | NPC1 like 1 | [Homo sapiens (human)] |
| 326 | APOB | 338 | apolipoprotein B | [Homo sapiens (human)] |
| 327 | HK2 | 3099 | hexokinase 2 | [Homo sapiens (human)] |
| 328 | STAR | 6770 | steroidogenic acute regulatory protein | [Homo sapiens (human)] |
| 329 | MIR200B | 406984 | microRNA 200b | [Homo sapiens (human)] |
| 330 | APOL1 | 8542 | apolipoprotein L1 | [Homo sapiens (human)] |
| 331 | MIR106B | 406900 | microRNA 106b | [Homo sapiens (human)] |
| 332 | MIR222 | 407007 | microRNA 222 | [Homo sapiens (human)] |
| 333 | SNCG | 6623 | synuclein gamma | [Homo sapiens (human)] |
| 334 | NME1 | 4830 | NME/NM23 nucleoside diphosphate kinase 1 | [Homo sapiens (human)] |
| 335 | ID1 | 3397 | inhibitor of DNA binding 1, HLH protein | [Homo sapiens (human)] |
| 336 | PTGIS | 5740 | prostaglandin I2 (prostacyclin) synthase | [Homo sapiens (human)] |
| 337 | SLC13A5 | 284111 | solute carrier family 13 member 5 | [Homo sapiens (human)] |
| 338 | GSTT1 | 2952 | glutathione S-transferase theta 1 | [Homo sapiens (human)] |
