# Supplementary material for: Molecular network-based analysis of the mechanism of liver injury induced by volatile oils from Artemisiae argyi folium
Source: BMC Complement Altern Med. 2017 Nov 16;17:491. doi: 10.1186/s12906-017-1997-4 (PMC5691807; doi:10.1186/s12906-017-1997-4)
Supplement: Supplementary file 2 — Target proteins of VOAAF searched in PubChem. Searched in the PubChem database, information on the drug targets of the VOAAF was listed. (DOCX 33 kb) [file 12906_2017_1997_MOESM2_ESM.docx]

**Table S2.** Target proteins of VOAAF searched in PubChem

| **No.** | **Target proteins** | **GI number** | **Species** |
| --- | --- | --- | --- |
| 1 | v-jun sarcoma virus 17 oncogene homolog (avian) [Homo sapiens] (gi: 119627033) | 119627033 | [Homo sapiens (human)] |
| 2 | 15-hydroxyprostaglandin dehydrogenase [NAD(+)] isoform 1 [Homo sapiens] (gi: 31542939) | 31542939 | [Homo sapiens (human)] |
| 3 | 15-lipoxygenase, partial [Homo sapiens] (gi: 1832253) | 1832253 | [Homo sapiens (human)] |
| 4 | 4-aminobutyrate aminotransferase, mitochondrial (gi: 48429239) | 48429239 | [Homo sapiens (human)] |
| 5 | 90-kda heat shock protein beta HSP90 beta [Homo sapiens] (gi: 4261762) | 4261762 | [Homo sapiens (human)] |
| 6 | activating transcription factor 6 [Homo sapiens] (gi: 119611100) | 119611100 | [Homo sapiens (human)] |
| 7 | ADAM10 [Homo sapiens] (gi: 2393947) | 2393947 | [Homo sapiens (human)] |
| 8 | aldehyde dehydrogenase 1 family, member A1 [Homo sapiens] (gi: 30582681) | 30582681 | [Homo sapiens (human)] |
| 9 | Aldo-keto reductase family 1 member B10 (gi: 322510010) | 322510010 | [Homo sapiens (human)] |
| 10 | Aldose reductase (gi: 113596) | 113596 | [Homo sapiens (human)] |
| 11 | Aldose reductase (gi: 1168407) | 1168407 | [Homo sapiens (human)] |
| 12 | Alkaline phosphatase, intestinal [Homo sapiens] (gi: 124376142) | 124376142 | [Homo sapiens (human)] |
| 13 | alkaline phosphatase, tissue-nonspecific isozyme isoform 1 precursor [Homo sapiens] (gi: 116734717) | 116734717 | [Homo sapiens (human)] |
| 14 | Androgen receptor (gi: 113830) | 113830 | [Homo sapiens (human)] |
| 15 | AR protein [Homo sapiens] (gi: 124375976) | 124375976 | [Homo sapiens (human)] |
| 16 | Arachidonate 5-lipoxygenase (gi: 126407) | 126407 | [Homo sapiens (human)] |
| 17 | Aromatase (gi: 117293) | 117293 | [Homo sapiens (human)] |
| 18 | aryl hydrocarbon receptor [Homo sapiens] (gi: 51095037) | 51095037 | [Homo sapiens (human)] |
| 19 | bcl-2-like protein 1 isoform 1 [Homo sapiens] (gi: 20336335) | 20336335 | [Homo sapiens (human)] |
| 20 | bcl-xL [Homo sapiens] (gi: 510901) | 510901 | [Homo sapiens (human)] |
| 21 | Beta-secretase 1 (gi: 296434407) | 296434407 | [Homo sapiens (human)] |
| 22 | Carbonic anhydrase 1 (gi: 115449) | 115449 | [Homo sapiens (human)] |
| 23 | Carbonic anhydrase 12 (gi: 5915866) | 5915866 | [Homo sapiens (human)] |
| 24 | Carbonic anhydrase 13 (gi: 30580350) | 30580350 | [Homo sapiens (human)] |
| 25 | Carbonic anhydrase 14 (gi: 8928036) | 8928036 | [Homo sapiens (human)] |
| 26 | Carbonic anhydrase 2 (gi: 115456) | 115456 | [Homo sapiens (human)] |
| 27 | Carbonic anhydrase 3 (gi: 134047703) | 134047703 | [Homo sapiens (human)] |
| 28 | Carbonic anhydrase 4 (gi: 115465) | 115465 | [Homo sapiens (human)] |
| 29 | Carbonic anhydrase 5A, mitochondrial (gi: 461680) | 461680 | [Homo sapiens (human)] |
| 30 | Carbonic anhydrase 5B, mitochondrial (gi: 8928041) | 8928041 | [Homo sapiens (human)] |
| 31 | Carbonic anhydrase 6 (gi: 116241278) | 116241278 | [Homo sapiens (human)] |
| 32 | Carbonic anhydrase 7 (gi: 1168744) | 1168744 | [Homo sapiens (human)] |
| 33 | Carbonic anhydrase 9 (gi: 83300925) | 83300925 | [Homo sapiens (human)] |
| 34 | carboxy-terminal domain RNA polymerase II polypeptide A small phosphatase 1 isoform 1 [Homo sapiens] (gi: 10864009) | 10864009 | [Homo sapiens (human)] |
| 35 | caspase recruitment domain family, member 15 [Homo sapiens] (gi: 119603173) | 119603173 | [Homo sapiens (human)] |
| 36 | Cellular tumor antigen p53 (gi: 269849759) | 269849759 | [Homo sapiens (human)] |
| 37 | Chain A, Crystal Structure Of The B1b2 Domains From Human Neuropilin-1 (gi: 160877737) | 160877737 | [Homo sapiens (human)] |
| 38 | Chain A, Crystal Structure Of The Human 2-Oxoglutarate Oxygenase Loc390245 (gi: 221046486) | 221046486 | [Homo sapiens (human)] |
| 39 | Chain A, Crystal Structure Of The Wild Type Ttr Binding Kaempferol (Ttrwt:kae) (gi: 422919337) | 422919337 | [Homo sapiens (human)] |
| 40 | Chain A, Human Ape1 Endonuclease With Bound Abasic Dna And Mn2+ Ion (gi: 6980812) | 6980812 | [Homo sapiens (human)] |
| 41 | Chain A, Human Complement Factor D In Complex With Isatoic Anhydride Inhibitor (gi: 157830332) | 157830332 | [Homo sapiens (human)] |
| 42 | Chain A, Jmjd2a Tandem Tudor Domains In Complex With A Trimethylated Histone H4-K20 Peptide (gi: 162330054) | 162330054 | [Homo sapiens (human)] |
| 43 | Chain A, Nucleophile Recognition As An Alternative Inhibition Mode For Benzoic Acid Based Carbonic Anhydrase Inhibitors (gi: 392935652) | 392935652 | [Homo sapiens (human)] |
| 44 | Chain A, The Structure Of Wild-Type Human Hadh2 (17beta-Hydroxysteroid Dehydrogenase Type 10) Bound To Nad+ At 1.2 A (gi: 122921310) | 122921310 | [Homo sapiens (human)] |
| 45 | Chain B, The Structure Of Wild-Type Human Hadh2 (17beta-Hydroxysteroid Dehydrogenase Type 10) Bound To Nad+ At 1.2 A (gi: 122921311) | 122921311 | [Homo sapiens (human)] |
| 46 | Cholinesterase (gi: 116353) | 116353 | [Homo sapiens (human)] |
| 47 | core-binding factor subunit beta isoform 1 [Homo sapiens] (gi: 13124881) | 13124881 | [Homo sapiens (human)] |
| 48 | Cyclin-dependent kinase 6 (gi: 266423) | 266423 | [Homo sapiens (human)] |
| 49 | cystic fibrosis transmembrane conductance regulator [Homo sapiens] (gi: 90421313) | 90421313 | [Homo sapiens (human)] |
| 50 | Cytochrome P450 1A1 (gi: 117139) | 117139 | [Homo sapiens (human)] |
| 51 | Cytochrome P450 1A2 (gi: 117144) | 117144 | [Homo sapiens (human)] |
| 52 | cytochrome P450 1A2 [Homo sapiens] (gi: 73915100) | 73915100 | [Homo sapiens (human)] |
| 53 | Cytochrome P450 1B1 (gi: 48429256) | 48429256 | [Homo sapiens (human)] |
| 54 | Cytochrome P450 2A6 (gi: 308153612) | 308153612 | [Homo sapiens (human)] |
| 55 | Cytochrome P450 2C19 (gi: 60416369) | 60416369 | [Homo sapiens (human)] |
| 56 | cytochrome P450 2C19 precursor [Homo sapiens] (gi: 4503219) | 4503219 | [Homo sapiens (human)] |
| 57 | Cytochrome P450 2C9 (gi: 6686268) | 6686268 | [Homo sapiens (human)] |
| 58 | cytochrome P450 2C9 precursor [Homo sapiens] (gi: 13699818) | 13699818 | [Homo sapiens (human)] |
| 59 | Cytochrome P450 2D6 (gi: 84028191) | 84028191 | [Homo sapiens (human)] |
| 60 | cytochrome P450 2D6 isoform 1 [Homo sapiens] (gi: 40805836) | 40805836 | [Homo sapiens (human)] |
| 61 | cytochrome P450 2D6 isoform 2 [Homo sapiens] (gi: 68509921) | 68509921 | [Homo sapiens (human)] |
| 62 | Cytochrome P450 3A4 (gi: 116241312) | 116241312 | [Homo sapiens (human)] |
| 63 | cytochrome P450 3A4 isoform 1 [Homo sapiens] (gi: 13435386) | 13435386 | [Homo sapiens (human)] |
| 64 | cytochrome P450, family 19, subfamily A, polypeptide 1, isoform CRA_a [Homo sapiens] (gi: 119597822) | 119597822 | [Homo sapiens (human)] |
| 65 | D(1A) dopamine receptor [Homo sapiens] (gi: 4503383) | 4503383 | [Homo sapiens (human)] |
| 66 | D(2) dopamine receptor isoform long [Homo sapiens] (gi: 4503385) | 4503385 | [Homo sapiens (human)] |
| 67 | Dihydrolipoamide dehydrogenase [Homo sapiens] (gi: 17391426) | 17391426 | [Homo sapiens (human)] |
| 68 | disintegrin and metalloproteinase domain-containing protein 17 preproprotein [Homo sapiens] (gi: 73747889) | 73747889 | [Homo sapiens (human)] |
| 69 | DNA polymerase beta [Homo sapiens] (gi: 4505931) | 4505931 | [Homo sapiens (human)] |
| 70 | DNA polymerase eta isoform 1 [Homo sapiens] (gi: 5729982) | 5729982 | [Homo sapiens (human)] |
| 71 | DNA polymerase iota [Homo sapiens] (gi: 154350220) | 154350220 | [Homo sapiens (human)] |
| 72 | DNA polymerase kappa [Homo sapiens] (gi: 7705344) | 7705344 | [Homo sapiens (human)] |
| 73 | Dual specificity protein kinase CLK1 (gi: 206729857) | 206729857 | [Homo sapiens (human)] |
| 74 | E3 ubiquitin-protein ligase UHRF1 isoform 1 [Homo sapiens] (gi: 115430235) | 115430235 | [Homo sapiens (human)] |
| 75 | ERAP1 protein [Homo sapiens] (gi: 21315078) | 21315078 | [Homo sapiens (human)] |
| 76 | ERAP2 protein [Homo sapiens] (gi: 40807029) | 40807029 | [Homo sapiens (human)] |
| 77 | Estradiol 17-beta-dehydrogenase 1 (gi: 313104233) | 313104233 | [Homo sapiens (human)] |
| 78 | Estradiol 17-beta-dehydrogenase 2 (gi: 544152) | 544152 | [Homo sapiens (human)] |
| 79 | estrogen nuclear receptor alpha [Homo sapiens] (gi: 348019627) | 348019627 | [Homo sapiens (human)] |
| 80 | euchromatic histone-lysine N-methyltransferase 2 [Homo sapiens] (gi: 168985070) | 168985070 | [Homo sapiens (human)] |
| 81 | FAD-linked sulfhydryl oxidase ALR [Homo sapiens] (gi: 54112432) | 54112432 | [Homo sapiens (human)] |
| 82 | Fatty acid-binding protein, adipocyte (gi: 119781) | 119781 | [Homo sapiens (human)] |
| 83 | Fatty acid-binding protein, epidermal (gi: 232081) | 232081 | [Homo sapiens (human)] |
| 84 | Fatty acid-binding protein, heart (gi: 119802) | 119802 | [Homo sapiens (human)] |
| 85 | Fatty acid-binding protein, intestinal (gi: 119805) | 119805 | [Homo sapiens (human)] |
| 86 | flap endonuclease 1 [Homo sapiens] (gi: 4758356) | 4758356 | [Homo sapiens (human)] |
| 87 | Free fatty acid receptor 1 (gi: 3122157) | 3122157 | [Homo sapiens (human)] |
| 88 | geminin [Homo sapiens] (gi: 7705682) | 7705682 | [Homo sapiens (human)] |
| 89 | GLS protein [Homo sapiens] (gi: 71051501) | 71051501 | [Homo sapiens (human)] |
| 90 | glucocorticoid receptor [Homo sapiens] (gi: 311348376) | 311348376 | [Homo sapiens (human)] |
| 91 | glutathione S-transferase omega-1 isoform 1 [Homo sapiens] (gi: 4758484) | 4758484 | [Homo sapiens (human)] |
| 92 | glycogen synthase kinase-3 beta isoform 1 [Homo sapiens] (gi: 21361340) | 21361340 | [Homo sapiens (human)] |
| 93 | GNAO1 protein [Homo sapiens] (gi: 34190601) | 34190601 | [Homo sapiens (human)] |
| 94 | Golgi-associated PDZ and coiled-coil motif-containing protein isoform b [Homo sapiens] (gi: 62868213) | 62868213 | [Homo sapiens (human)] |
| 95 | guanine nucleotide-binding protein G(i) subunit alpha-1 isoform 1 [Homo sapiens] (gi: 33946324) | 33946324 | [Homo sapiens (human)] |
| 96 | guanine nucleotide-binding protein G(o) subunit alpha isoform a [Homo sapiens] (gi: 10567816) | 10567816 | [Homo sapiens (human)] |
| 97 | heat shock 70kDa protein 5 (glucose-regulated protein, 78kDa) [Homo sapiens] (gi: 168984549) | 168984549 | [Homo sapiens (human)] |
| 98 | heat shock protein beta-1 [Homo sapiens] (gi: 4504517) | 4504517 | [Homo sapiens (human)] |
| 99 | heat shock protein HSP 90-alpha isoform 2 [Homo sapiens] (gi: 154146191) | 154146191 | [Homo sapiens (human)] |
| 100 | histone acetyltransferase KAT2A [Homo sapiens] (gi: 153791535) | 153791535 | [Homo sapiens (human)] |
| 101 | huntingtin [Homo sapiens] (gi: 90903231) | 90903231 | [Homo sapiens (human)] |
| 102 | hypoxia-inducible factor 1, alpha subunit (basic helix-loop-helix transcription factor) [Homo sapiens] (gi: 32879895) | 32879895 | [Homo sapiens (human)] |
| 103 | integrin alpha-4 precursor [Homo sapiens] (gi: 67191027) | 67191027 | [Homo sapiens (human)] |
| 104 | interleukin 8 [Homo sapiens] (gi: 186368) | 186368 | [Homo sapiens (human)] |
| 105 | kallikrein-7 isoform 1 preproprotein [Homo sapiens] (gi: 21327705) | 21327705 | [Homo sapiens (human)] |
| 106 | Lactoylglutathione lyase (gi: 134039205) | 134039205 | [Homo sapiens (human)] |
| 107 | lens epithelium-derived growth factor p75 [Homo sapiens] (gi: 6708281) | 6708281 | [Homo sapiens (human)] |
| 108 | lysosomal alpha-glucosidase preproprotein [Homo sapiens] (gi: 119393891) | 119393891 | [Homo sapiens (human)] |
| 109 | Matrix metalloproteinase-9 (gi: 269849668) | 269849668 | [Homo sapiens (human)] |
| 110 | melanocortin receptor 4 [Homo sapiens] (gi: 119508433) | 119508433 | [Homo sapiens (human)] |
| 111 | Methyl-CpG binding domain protein 2 [Homo sapiens] (gi: 21595776) | 21595776 | [Homo sapiens (human)] |
| 112 | Microtubule-associated protein tau [Homo sapiens] (gi: 92096784) | 92096784 | [Homo sapiens (human)] |
| 113 | MPI protein [Homo sapiens] (gi: 16878311) | 16878311 | [Homo sapiens (human)] |
| 114 | muscarinic acetylcholine receptor M1 [Homo sapiens] (gi: 37622910) | 37622910 | [Homo sapiens (human)] |
| 115 | muscarinic acetylcholine receptor M5 [Homo sapiens] (gi: 7108336) | 7108336 | [Homo sapiens (human)] |
| 116 | Myeloid cell leukemia sequence 1 (BCL2-related) [Homo sapiens] (gi: 78070770) | 78070770 | [Homo sapiens (human)] |
| 117 | NFKB1 protein, partial [Homo sapiens] (gi: 21620132) | 21620132 | [Homo sapiens (human)] |
| 118 | Nrf2 [Homo sapiens] (gi: 693842) | 693842 | [Homo sapiens (human)] |
| 119 | nuclear factor erythroid 2-related factor 2 isoform 1 [Homo sapiens] (gi: 20149576) | 20149576 | [Homo sapiens (human)] |
| 120 | nuclear receptor coactivator 1 isoform 1 [Homo sapiens] (gi: 22538455) | 22538455 | [Homo sapiens (human)] |
| 121 | nuclear receptor coactivator 3 isoform a [Homo sapiens] (gi: 32307126) | 32307126 | [Homo sapiens (human)] |
| 122 | Nuclear receptor ROR-gamma (gi: 49066040) | 49066040 | [Homo sapiens (human)] |
| 123 | nuclear receptor subfamily 0 group B member 1 [Homo sapiens] (gi: 5016090) | 5016090 | [Homo sapiens (human)] |
| 124 | nuclear receptor subfamily 5 group A member 2 isoform 2 [Homo sapiens] (gi: 4504343) | 4504343 | [Homo sapiens (human)] |
| 125 | peripheral myelin protein 22 [Rattus norvegicus] (gi: 8393992) | 8393992 | [Homo sapiens (human)] |
| 126 | peroxisome proliferator activated receptor gamma [Homo sapiens] (gi: 216409692) | 216409692 | [Homo sapiens (human)] |
| 127 | Peroxisome proliferator-activated receptor alpha (gi: 3041727) | 3041727 | [Homo sapiens (human)] |
| 128 | Peroxisome proliferator-activated receptor delta (gi: 417522) | 417522 | [Homo sapiens (human)] |
| 129 | Peroxisome proliferator-activated receptor gamma (gi: 13432234) | 13432234 | [Homo sapiens (human)] |
| 130 | Phosphatidylinositol 5-phosphate 4-kinase type-2 alpha (gi: 18266879) | 18266879 | [Homo sapiens (human)] |
| 131 | platelet-activating factor acetylhydrolase IB subunit beta isoform b [Homo sapiens] (gi: 296080766) | 296080766 | [Homo sapiens (human)] |
| 132 | platelet-activating factor acetylhydrolase IB subunit gamma [Homo sapiens] (gi: 225543099) | 225543099 | [Homo sapiens (human)] |
| 133 | pregnane X nuclear receptor [Homo sapiens] (gi: 325495557) | 325495557 | [Homo sapiens (human)] |
| 134 | PRMT1 protein, partial [Homo sapiens] (gi: 32425330) | 32425330 | [Homo sapiens (human)] |
| 135 | prostaglandin E2 receptor EP2 subtype [Homo sapiens] (gi: 31881630) | 31881630 | [Homo sapiens (human)] |
| 136 | rac GTPase-activating protein 1 [Homo sapiens] (gi: 21361397) | 21361397 | [Homo sapiens (human)] |
| 137 | receptor-interacting serine/threonine-protein kinase 2 [Homo sapiens] (gi: 4506537) | 4506537 | [Homo sapiens (human)] |
| 138 | Receptor-type tyrosine-protein kinase FLT3 (gi: 156630887) | 156630887 | [Homo sapiens (human)] |
| 139 | retinoic acid nuclear receptor alpha variant 1 [Homo sapiens] (gi: 325495463) | 325495463 | [Homo sapiens (human)] |
| 140 | retinoid X nuclear receptor alpha [Homo sapiens] (gi: 325495497) | 325495497 | [Homo sapiens (human)] |
| 141 | RGS12 [Homo sapiens] (gi: 3290016) | 3290016 | [Homo sapiens (human)] |
| 142 | RGS8 protein [Homo sapiens] (gi: 74355113) | 74355113 | [Homo sapiens (human)] |
| 143 | runt-related transcription factor 1 isoform AML1c [Homo sapiens] (gi: 19923198) | 19923198 | [Homo sapiens (human)] |
| 144 | sentrin-specific protease 8 [Homo sapiens] (gi: 262118306) | 262118306 | [Homo sapiens (human)] |
| 145 | serine/threonine-protein kinase 16 [Homo sapiens] (gi: 57165436) | 57165436 | [Homo sapiens (human)] |
| 146 | serine/threonine-protein kinase pim-2 [Homo sapiens] (gi: 42821112) | 42821112 | [Homo sapiens (human)] |
| 147 | Sialate O-acetylesterase (gi: 74734243) | 74734243 | [Homo sapiens (human)] |
| 148 | Steroid hormone receptor ERR1 (gi: 215274146) | 215274146 | [Homo sapiens (human)] |
| 149 | Succinate-semialdehyde dehydrogenase, mitochondrial (gi: 7531278) | 7531278 | [Homo sapiens (human)] |
| 150 | TDP1 protein [Homo sapiens] (gi: 79154014) | 79154014 | [Homo sapiens (human)] |
| 151 | Thrombopoietin [Homo sapiens] (gi: 120660324) | 120660324 | [Homo sapiens (human)] |
| 152 | Tissue factor (gi: 135666) | 135666 | [Homo sapiens (human)] |
| 153 | toll-like receptor 9 [Homo sapiens] (gi: 194068499) | 194068499 | [Homo sapiens (human)] |
| 154 | Transient receptor potential cation channel subfamily A member 1 (gi: 313104269) | 313104269 | [Homo sapiens (human)] |
| 155 | troponin C, slow skeletal and cardiac muscles [Homo sapiens] (gi: 4507615) | 4507615 | [Homo sapiens (human)] |
| 156 | troponin I, cardiac muscle [Homo sapiens] (gi: 151101270) | 151101270 | [Homo sapiens (human)] |
| 157 | troponin T, cardiac muscle isoform 3 [Homo sapiens] (gi: 48255881) | 48255881 | [Homo sapiens (human)] |
| 158 | Tyrosine-protein phosphatase non-receptor type 1 (gi: 131467) | 131467 | [Homo sapiens (human)] |
| 159 | UDP-glucuronosyltransferase 2B7 (gi: 136727) | 136727 | [Homo sapiens (human)] |
